# Diversity of Dysregulated Long Non-Coding RNAs in HBV-Related Hepatocellular Carcinoma

**DOI:** 10.3389/fimmu.2022.834650

**Published:** 2022-01-28

**Authors:** Nazia Samudh, Creanne Shrilall, Patrick Arbuthnot, Kristie Bloom, Abdullah Ely

**Affiliations:** Wits/South African Medical Research Council (SAMRC) Antiviral Gene Therapy Research Unit, School of Pathology, Faculty of Health Sciences, University of the Witwatersrand, Johannesburg, South Africa

**Keywords:** HBV-related HCC, lncRNA, epigenetic regulation, transcriptional regulation, ceRNA, protein interactions, miRNA precursor processing

## Abstract

Infection with the hepatitis B virus (HBV) continues to pose a major threat to public health as approximately 292 million people worldwide are currently living with the chronic form of the disease, for which treatment is non-curative. Chronic HBV infections often progress to hepatocellular carcinoma (HCC) which is one of the world’s leading causes of cancer-related deaths. Although the process of hepatocarcinogenesis is multifaceted and has yet to be fully elucidated, several studies have implicated numerous long non-coding RNAs (lncRNAs) as contributors to the development of HCC. These host-derived lncRNAs, which are often dysregulated as a consequence of viral infection, have been shown to function as signals, decoys, guides, or scaffolds, to modulate gene expression at epigenetic, transcriptional, post-transcriptional and even post-translational levels. These lncRNAs mainly function to promote HBV replication and oncogene expression or downregulate tumor suppressors. Very few lncRNAs are known to suppress tumorigenesis and these are often downregulated in HCC. In this review, we describe the mechanisms by which lncRNA dysregulation in HBV-related HCC promotes tumorigenesis and cancer progression.

## 1 Introduction

Once thought to be transcriptional noise by virtue of their apparent inability to encode proteins, long non-coding RNAs (lncRNAs) have since emerged as vital regulators of gene expression. The discovery and annotation of lncRNAs have picked up speed over the last few years, thanks to availability of deep sequencing techniques and bioinformatic tools. LncRNAs are larger than 200 bases, a feature that distinguishes them from other non-coding RNAs, although they may vary in size from a few hundred to a few thousand nucleotides. These diverse molecules are encoded at various regions of the genome and can be classified according to their orientation in relation to the nearby protein coding genes [reviewed in (1)]. Long intergenic non-coding RNAs, or lincRNAs, are the most abundant type of lncRNA and, as the name suggests, are encoded in the vast expanse of DNA that separates protein coding regions. The second most abundant type of lncRNA are the antisense lncRNAs which are transcribed from the opposite strand of protein coding genes. Thus, these lncRNAs possess the inherent ability to interact through complementary base pairing with mRNA transcribed from the same location and thereby regulate it. Less abundant sense lncRNAs are encoded within protein-coding genes in the same orientation as the gene, and intronic lncRNAs can be found within the introns of genes. Finally, the least abundant are bi-directional lncRNAs, which are located adjacent to protein-coding genes and are transcribed from the same promoter, but in an opposite direction. LncRNAs may act in *cis* on nearby genes or in *trans* on distant genes, mRNA or proteins (2). Like mRNAs, lncRNAs are transcribed by RNA polymerase II and may contain 5’ caps, polyadenylated tails, multiple exons, and can also undergo alternative splicing albeit to a lesser extent [reviewed in (2)].

## 2 Hepatitis B Virus (HBV)-Related Hepatocellular Carcinoma (HCC) and LncRNA Dysregulation

The development of lncRNA microarrays has facilitated comparative profiling of lncRNAs in cancerous and non-cancerous tissues. Numerous lncRNAs are now known to display differential expression profiles in most types of cancers, including HBV-related HCC [reviewed in (3–5)]. Chronic HBV infection is a major risk factor for development of HCC. Prolonged HBV replication promotes immune-mediated inflammation of the liver which results in cirrhosis and eventual malignant transformation of hepatocytes (6). In a previous review, we discussed the roles of several key lncRNAs in the etiology of HBV-related HCC (4). Subsequent studies have further characterized several of these lncRNAs providing valuable insight into their functionality, and potential as therapeutic targets or biomarkers. In addition, many other lncRNAs have been found to be upregulated during chronic HBV infection and have been implicated in progression to HCC. An updated summary of lncRNAs dysregulated in HBV-related HCC and their functions can be found in **Table 1**.

**Table 1:** LncRNAs dysregulated in HBV-related HCC and their functions.

LncRNA	Dysregulation	Mechanism of Action	Role in HBV-related HCC	References
HEIH	Upregulated	EZH2-mediated silencing of p15, p16, p21 and p57	Cell proliferation	(7)
UCA1	Upregulated	EZH2-mediated silencing of p27	Cell proliferation	(8)
Linc00152	Upregulated	EZH2-mediated silencing of *E-cadherin* Enhances promoter activity of *EpCAM*	Epithelial-mesenchymal transition (EMT) Stemness, tumorigenesis, cell proliferation	(9, 10)
PVT1	Upregulated	Negatively regulates expression of EZH2 and binds to EZH2 to prevent formation of the PRC2 complex and its recruitment to the *MYC* promoter	Cell proliferation, migration, and invasion	(11)
DLEU2	Upregulated	Relieves EZH2 suppression of cccDNA	Promotes viral replication	(12)
HOTTIP	Upregulated	WDR5/MKK-mediated activation of *HOXA13*	Suppresses viral replication	(13)
HOTAIR	Upregulated	Functions as a scaffold to facilitate ubiquitination of SUZ12 and ZNF198 resulting in destabilization of repressive chromatin modifying complexes. Recruits transcription factor Sp1 to HBV promoter	Promotes viral replication, and pluripotency of hepatocytes Promotes viral replication	(14–16)
HULC	Upregulated	Stimulates HBx to activate and recruit STAT3 to the miR-539 promoter. miR-539 post-transcriptionally silences APOBEC3B Represses transcription of p18 Sequesters miR-372 leading to de-repression of PRKACB which activates CREB	Stabilizes cccDNA and promotes HBV replication Cell proliferation Upregulates expression of HULC	(17–19)
MALAT1	Upregulated	Recruits Sp1 to the promoter of the *LTBP3* gene	EMT	(20)
Linc01152	Upregulated	Binds to IL-23 promoter and enhances transcription of IL-23 which promotes activation of STAT3	Th17-induced liver damage Cell proliferation	(21, 22)
ZEB2-AS1	Upregulated	Alternative splicing of ZEB2-encoding mRNA, increases translation of ZEB2 which is a transcriptional repressor of E-cadherin	EMT	(23)
HBx-LINE1	Upregulated	Sequesters miR-122, decreases E-cadherin and increases WNT1	EMT Activates Wnt/β-catenin signaling pathway	(24, 25)
DBH-AS1	Upregulated	Promotes MAPK signaling by unknown mechanism Sequesters miR-138 leading to activation of the FAK/Src/ERK pathway	Cell proliferation Cell proliferation EMT	(26, 27)
PCNAP1	Upregulated	Sequesters miR-154 which prevents it from inhibiting PCNA, thus allowing PCNA to bind cccDNA Sequesters miR-340-5p and prevents it from repressing ATF7	Promotes HBV replication and cccDNA accumulation Cell proliferation	(28, 29)
WEE2-AS1	Upregulated	May sequester miR-214 and inhibit post-transcriptional silencing of FERMT3	Cell proliferation EMT Hepatic vascular invasion Decreased apoptosis	(30, 31)
Unigene 56159	Upregulated	Sequesters miR-140-5p and prevents it from repressing Slug which is a repressor of E-cadherin	EMT	(32, 33)
H19	Upregulated Downregulated	Sequesters miR-22 to increase the expression of EMT-associated proteins Encodes miR-675 thereby inhibiting PPARα which then promotes Akt/mTOR signalling Recruits histone acetylase HnRNP U/PCAF/RNA PolII protein complex for epigenetic activation of miR-200 family	EMT Cell proliferation HBx-induced cell damage Inhibits EMT	(34–36)
n335586	Upregulated	Sequesters miR-924 thereby inhibiting its repressive activity on CKMT1A	EMT	(37)
DREH/hDREH (human ortholog)	Downregulated	Binds to and alters the structure of vimentin to inhibit metastasis	Downregulation of DREH increases cell proliferation and EMT	(38, 39)
SAMD12-AS1	Upregulated	Interacts with NMP1 and prevents it from binding with E3 ligase HDM2, thus allowing HDM2 to bind to and enhance the degradation of p53	Cell proliferation Inhibits apoptosis	(40)
HUR1	Upregulated	Binds to p53 thereby inhibiting its transcriptional regulation on p21 and Bax	Cell proliferation Tumor progression	(41)
MVIH	Upregulated	Interacts with PGK1 and inhibits its secretion	Angiogenesis	(42)
LncRNA-6195	Downregulated	Binds to ENO1 and inhibits its enzymatic activity	Downregulation of lncRNA-6195 promotes tumor growth and energy metabolism	(43)
Ftx	Upregulated	Precursor for the miR-545/374a cluster	Cell proliferation EMT	(44, 45)

Certain lncRNAs, such as UCA1, DLEU2, HULC and DBH-AS1, can be directly or indirectly upregulated by the HBV x (HBx) protein (8, 12, 18, 26). However, these lncRNAs may not necessarily be specific to HBV-related HCC as several, including those not induced by HBx, are commonly dysregulated in a variety of other cancers (5). Differential lncRNA plasma concentrations are also observed in individuals with resolved, inactive, or chronic HBV infection (46). Certain lncRNAs, such as HOTTIP, MEG3 and PCAT32, are significantly upregulated in resolved cases, and may serve as early prognostic markers for resolution of HBV infection.

Whilst a few dysfunctional lncRNAs are common to HCCs of different viral etiologies, most are specific to the type of virus-induced HCC [reviewed in (4, 47)]. Since hepatitis virus-induced HCCs account for most cases, lncRNAs that are dysregulated in non-viral HCCs have not been fully investigated. A recent study shows that non-viral HCC also exhibits a differential expression profile when compared to hepatitis virus-induced HCC (48). However, lncRNAs such as HOTAIR and H19 have been found to be upregulated in obesity-induced HCC (49). These lncRNAs, along with MALAT1 and HULC, are also upregulated in non-alcoholic fatty liver disease which is a risk factor for HCC (50). Additional studies, taking HCC etiology into consideration, are required to determine which lncRNAs are commonly dysregulated.

LncRNAs are instrumental in the proliferation, metastasis, and invasion of cancerous cells, tumor angiogenesis, covalently closed circular DNA (cccDNA) stabilization, and modulation of HBV replication [reviewed in (3, 40, 51)]. Thus, lncRNAs have the potential to serve as novel therapeutic targets. To this end, understanding the mechanism of action is crucial to developing relevant therapies. Numerous studies have been conducted with the aim of elucidating the role of lncRNAs in various cancers. What can be gathered is that a single lncRNA can interact with several biomolecules to initiate or promote oncogenesis. This review will describe the mechanisms of action of a variety of dysregulated lncRNAs associated with HBV-related HCC.

## 3 Mechanisms of Action of LncRNAs

The molecular interactions between lncRNAs and their targets are quite diverse. Although not entirely clear, these interactions are most likely to be dependent on the sequence of the lncRNA. These sequences would influence DNA or RNA interactions through complementary base pairing, or the secondary structure of lncRNAs, which may enable interactions with target proteins [reviewed in (52)]. These molecular interactions are classified into four archetypes: scaffold, guide, decoy and signal [reviewed in (53)]. LncRNAs which form scaffolds support the formation and stabilization of protein complexes whereas guide lncRNAs direct their targets to specific regions of the genome. LncRNAs can also mimic the targets of transcription factors and microRNAs (miRNAs) and thus act as decoys to regulate gene expression. Signal lncRNAs are transcribed at precise stages of cell development and may act as signals for transcription factors to initiate transcription of developmental genes. These archetypes are not mutually exclusive as certain lncRNAs can exert gene regulatory functions by more than one type of molecular interaction.

The interacting partners of lncRNAs and the mechanism by which lncRNAs exert their functions largely depends on their cellular location [reviewed in (3)]. LncRNAs located in the nuclear compartment interact mainly with DNA or proteins to regulate gene expression at an epigenetic or translational level. Some nuclear lncRNAs interact with RNA and proteins to alter splicing patterns. Cellular lncRNAs can interact with RNA or proteins to regulate gene expression post-transcriptionally or post-translationally respectively. Post-transcriptional regulation is mediated by lncRNAs acting as competitive endogenous RNAs (ceRNAs) which sequester or sponge miRNAs to de-repress miRNA-mediated post-transcriptional silencing of certain genes. On the other hand, certain lncRNAs are precursors of miRNAs and can silence mRNA expression. LncRNAs can directly bind to and stabilize proteins, enhancing their bioavailability and functionality. Conversely, certain lncRNAs can also alter or inhibit the functionality of proteins. LncRNAs can also promote protein degradation by facilitating ubiquitination (40). The mechanisms by which HBV-specific lncRNAs exert their oncogenic functions are described below.

### 3.1 Transcriptional Regulation in the Nucleus

**Figure 1:**
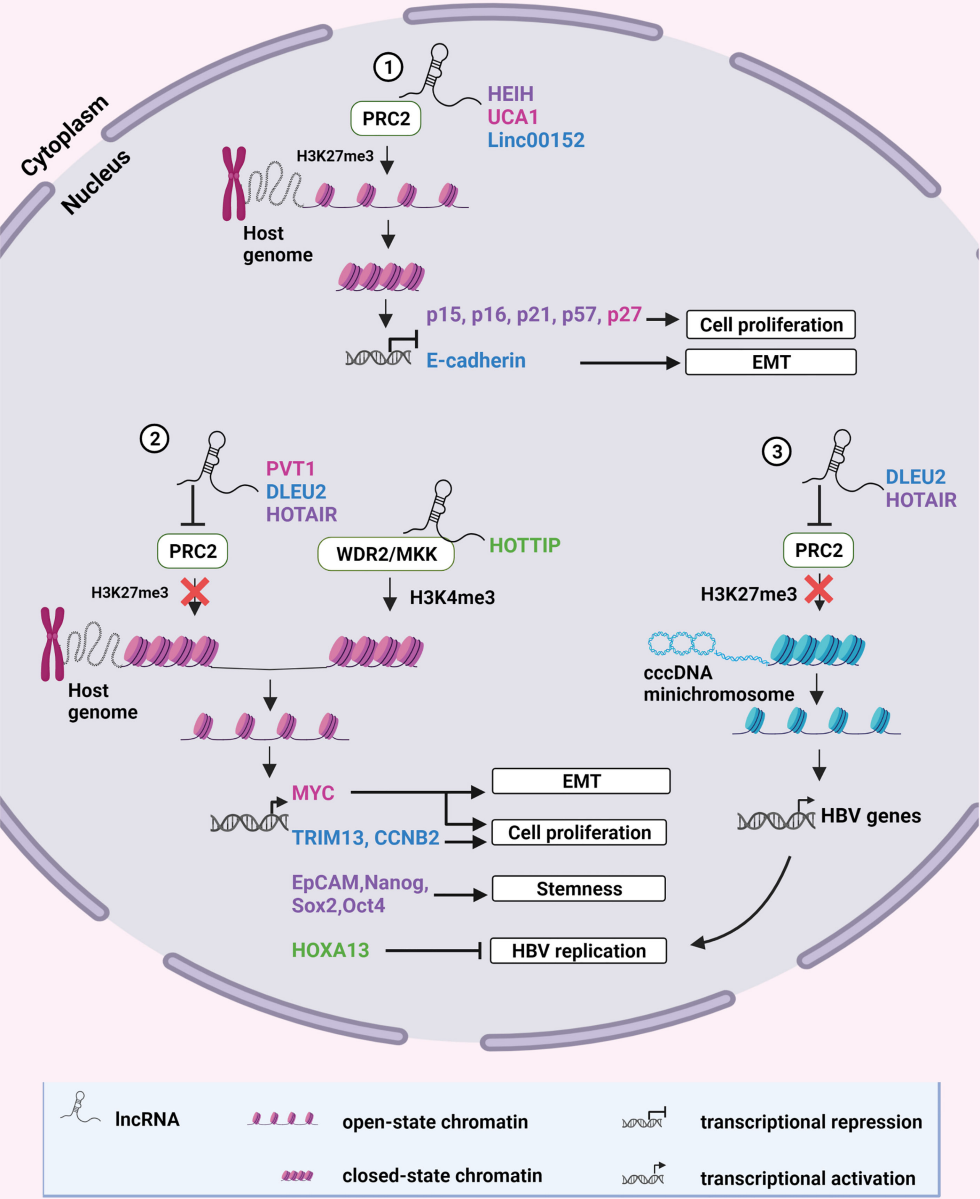
Epigenetic modification of chromatin by lncRNAs. **(1)** LncRNAs HEIH, UCA1 and Linc00152 recruit the repressive chromatin modifying complex, PRC2, to specific sites on the human genome to catalyze H3K27 trimethylation. This results in chromatin compaction and transcriptional repression of tumor suppressor genes. **(2)** LncRNAs PVT1 and HOTAIR inhibit formation of the PRC2 complex whereas DLEU2 displaces PRC2 from chromatin to prevent H3K27 methylation, allowing transcription of oncogenic host genes. HOTTIP recruits the activating chromatin-modifying complex WDR2/MKK to mediate H3K4 methylation of chromatin enabling the transcription of HOXA13 which suppresses HBV replication. **(3)** DLEU2 and HOTAIR can also inhibit PRC2 recruitment to HBV cccDNA, alleviating epigenetic silencing and promoting HBV replication. Color coding of names indicate a functional relationship between the lncRNA and its target. Created with BioRender.com.

Oncogenes can be upregulated, or tumor suppressor genes downregulated by epigenetic modification of chromatin, which alters the accessibility of the DNA to transcription factors and proteins. Up to 38% of lincRNAs alter chromatin architecture, mainly by directing repressive chromatin modifying complexes to specific genomic sites (54). The Polycomb Repressor complex 2 (PRC2) is one such repressive chromatin modifying complex that is commonly modulated by HBV-dysregulated lncRNAs. PRC2 consists of three core subunits: the Enhancer of Zest Homolog 2 (EZH2), the Suppressor of Zest homolog 12 (SUZ12), and the Embryonic Ectoderm Development (EED) subunits (55). SUZ12 is responsible for the structural stability of the complex. EED guides the PRC2 to histone 3 (H3) lysine 27 (K27) and stimulates the methyltransferase activity of EZH2, which catalyzes the tri-methylation of H3K27 (H3K27me3). This action subsequently leads to chromatin compaction and transcriptional repression of the targeted genes, which are usually involved in the regulation of cell differentiation, development and fate during embryogenesis (56). Conversely, lncRNAs can promote open chromatin structure by preventing PRC2-mediated compaction or by recruiting activating chromatin modifiers to the sites of certain viral or host oncogenes, which can then be transcribed (**Figure 1**).

**Figure 2:**
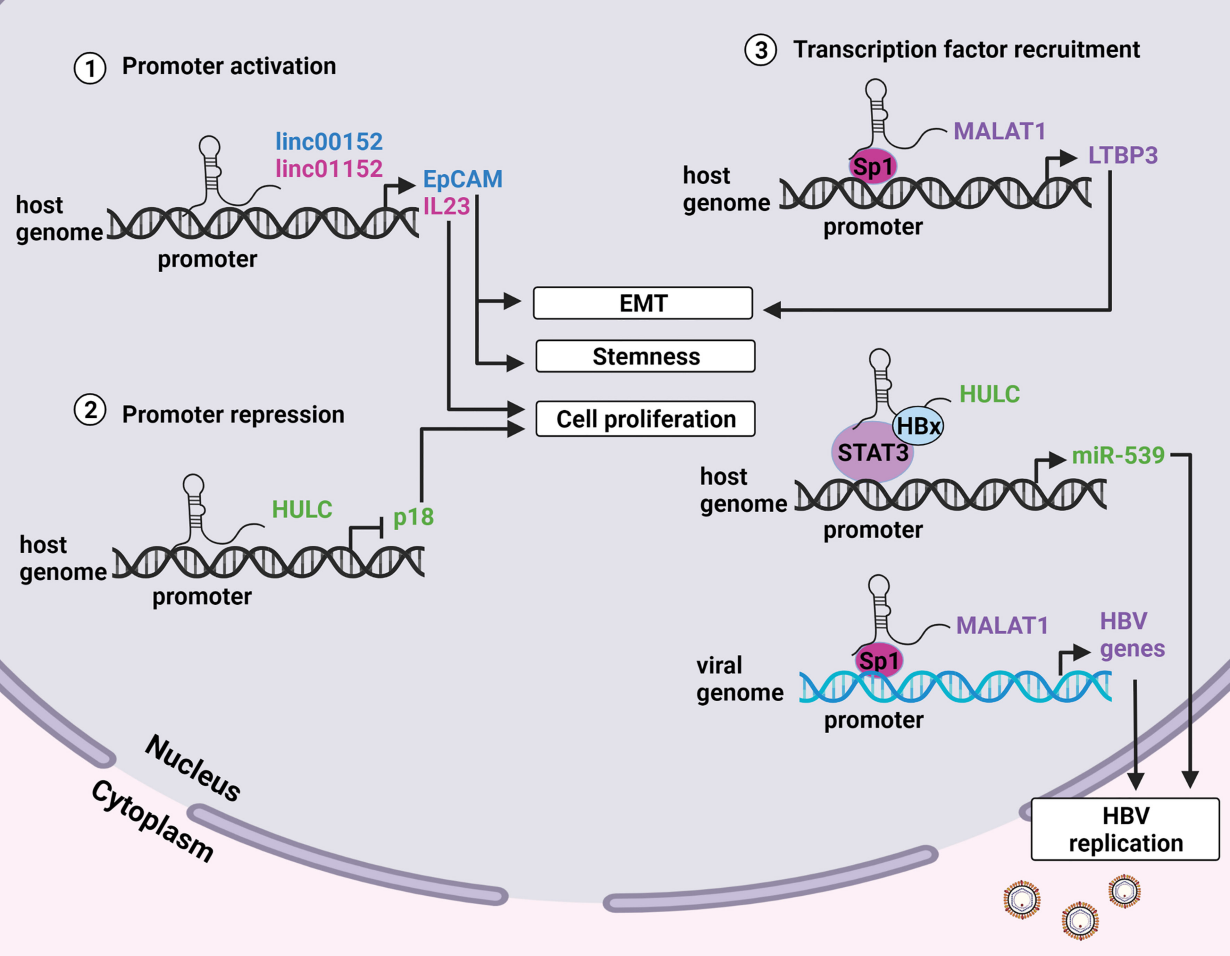
LncRNA modulation of promoter activity. **(1)** LncRNAs linc00152 and linc01152 have enhancer-like properties and interact with the promoters of the EpCAM and IL23 genes respectively. EpCAM positive cells display enhanced stemness and metastatic potential, and increased levels of IL23 promote proliferation. **(2)** LncRNA HULC interacts with the promoter of the tumor suppressor p18 to inhibit its transcription and promote cell proliferation. **(3)** Certain lncRNAs can guide transcription factors to the promoters of specific genes to enhance transcription. MALAT1 recruits the transcription factor Sp1 to the promoter of the LTBP3 gene on the host genome resulting in metastasis. HULC and HOTAIR recruit the transcription factors STAT3 and Sp1 to host and viral genomes respectively to enhance transcription of viral genes thus promoting HBV replication. Created with BioRender.com.

Transcription of genes can also be enhanced or suppressed by modulating the activities of transcription factors that interact with the promoter regions of a gene. Certain lncRNAs regulate gene expression by direct interaction with the promoters of protein coding genes or by recruiting transcription factors to promoter sites (**Figure 2**) [reviewed in (51)]. The lncRNAs which employ epigenetic and/or direct transcriptional gene regulatory mechanisms to promote HBV replication, persistence and HCC are described below.

#### 3.1.1 HEIH

High expression in hepatocellular carcinoma (HEIH) is a 1,781 nucleotide long, polyadenylated lincRNA that was first identified in HBV-related HCC tissues (7). Its transcription is upregulated by the transcription factor Sp1 in HCC and HBV-infected cirrhotic tissue specimens. HEIH associates with the EZH2 subunit of the PRC2 through its 5’ end and recruits EZH2 to the promoter regions of the cell-cycle regulatory proteins p15, p16, p21 and p57. These genes are subsequently silenced, leading to increased cell proliferation and ultimately HCC. Levels of HEIH are also known to increase as disease progresses from a chronic HBV infection to cirrhosis then HCC, and could be useful to estimate the stage of disease (57)

#### 3.1.2 UCA1

Urothelial Cancer associated 1 (UCA1) is a 1,400 nucleotide long lincRNA that was first identified as a marker of bladder transitional cell carcinoma (58). It was also found to be upregulated by HBx in HBV-related HCC specimens and HBx-expressing cell lines (8). Its expression is associated with increased cell proliferation and inhibition of apoptosis. As with many other lincRNAs, UCA1 can recruit EZH2 to mediate epigenetic silencing. Its target is the tumor suppressor p27, which when suppressed, allows cyclin-dependent kinase 2 (CDK2) to accelerate G1/S cell cycle progression, cell proliferation and development of HCC.

#### 3.1.3 Linc00152

Linc00152 is upregulated by HBx in HBV-related HCC tissues where it is localized mainly in the nucleus and promotes epithelial-mesenchymal transition (EMT) (9). The hallmark of EMT is switching from E-cadherin to N-cadherin expression, which increases cellular metastasis and invasiveness, and is associated with an overall poor patient outcome in many types of cancer [reviewed in (59)]. At an epigenetic level, linc00152 recruits EZH2 to the promoter of the gene encoding E-cadherin to induce its suppression, and promote EMT (9). As a *cis*-acting lincRNA in HCC-related tissues, linc00152 also enhances the promoter activity of the nearby *Epithelial Cell Adhesion Molecule (EpCAM)* gene, which results in the activation of mTOR signaling (10). EpCAM-positive HCC cells display stem cell-like properties, which promote tumor formation and proliferation (60). *In vitro* knockdown of linc00152 in HCC cells decreases the promoter activity of *EpCAM*, and increases expression of E-cadherin while suppressing N-cadherin to successfully decrease the proliferative and migratory capacity of HCC cells (9, 10). Targeting this lincRNA may reduce the likelihood of metastasis in HBV-related HCC.

#### 3.1.4 PVT1

Plasmacytoma Variant Translocation 1 (PVT1) is a 1,716 nucleotide long lncRNA that is progressively upregulated by TGF-β1 in HBV-related HCC (11, 61). PVT1 alleviates PRC2-mediated repression of the downstream *MYC* oncogenes *via* two mechanisms (11). Firstly, it negatively regulates the expression of EZH2 to reduce its concentration in the nucleus, although the exact mechanism by which this occurs has not been determined. Secondly, it directly binds to EZH2 to prevent formation of the PRC2 complex and its recruitment to the *MYC* promoter. The resultant hypomethylation of this region results in active transcription of *c-myc* and promotes cell proliferation, migration, and invasion by HBV-positive Hep3B liver cell lines.

#### 3.1.5 DLEU2

Deleted in Lymphocytic Leukemia 2 (DLEU2) is a large antisense lncRNA consisting of 15 exons which can be alternatively spliced to yield three isoforms, the largest of which partially overlaps the *RFP2/LEU5* gene (62). It is known to function as a tumor suppressor gene since its deletion was associated with the development of chronic lymphocytic leukemia (63). However, the role of DLEU2 in HCC is quite the opposite. In cellular models of HBV infection, HBx was recently shown to directly promote the expression of DLEU2 by binding to its promoter (12). In addition to upregulating this lncRNA, HBx also directly interacts with DLEU2 at partially overlapping binding sites that are shared with EZH2 (12). Recruitment of HBx-DLEU2 to the viral cccDNA minichromosome thus interferes with EZH2-mediated repression of viral genes to increase viral replication. Similarly, HBx also recruits DLEU2 to host promoter sequences to relieve transcriptional repression of selected host genes, such as TRIM13 and CCNB2. This is achieved by displacing EZH2/PRC2 complexes.

#### 3.1.6 HOTTIP

HOXA transcript at the distal tip (HOTTIP) is a 3,764 nucleotide long antisense lincRNA that is located at the 5’ terminus of the HOXA locus (64). As mentioned earlier, HOTTIP is upregulated in cases of resolved HBV and may thus have an important role in promoting the clearance of HBV. Its expression is also upregulated in an HBx-independent manner in HBV-related HCC tissues where it plays a role in suppressing HBV replication (13). In this instance, it is the HBV DNA polymerase which stabilizes the cAMP-responsive element binding (CREB) transcription factor to promote the expression of HOTTIP. HOTTIP then recruits the chromatin modifying complex WDR5/MKK to the HOXA gene locus through chromosomal looping to mediate H3K4 trimethylation and transcriptional activation of the downstream HOXA13 gene (64). HOXA13 thereafter binds to the HBV enhancer 1 and X promoter to restrain viral replication. In this manner, HBV polymerase negatively regulates replication, possibly to avoid host immune responses, and establish chronicity [reviewed in (65)].

Recently, HOTTIP was found to be significantly upregulated in exosomes obtained from the sera of chronically infected HBV patients who were treated with the nucleoside analog Tenofovir Alafenamide (TAF) (66). The exosomal presence of HOTTIP seemed to enhance the antiviral effects of TAF as evidenced by the significantly downregulated levels of HBV surface antigen (HBsAg) and HBV e antigen (HBeAg), and most importantly cccDNA in HepAD38 cells treated with patient derived exosomes. These promising results were observed in a small study and still require further *in vivo* investigation.

In non-HBV-related HCC specimens, upregulation of HOTTIP, and the associated increase in HOXA13, is an indication of metastatic potential, increased probability of disease recurrence following liver transplantation, and poor overall survival (67, 68). Upregulation of HOTTIP and HOXA13 was also confirmed in several HCC-derived cell lines. Thus, even though HOXA13 suppresses HBV replication, it may also play a role in promoting HBV-related HCC, although the exact mechanism is yet to be examined.

#### 3.1.7 HOTAIR

HOX Transcript Antisense RNA (HOTAIR) is an approximately 2,200 nucleotide long lincRNA located in the HOXC locus (69). Under normal physiological conditions, HOTAIR acts as a scaffold to simultaneously bind PRC2 and a second repressive chromatin-modifying complex: the LSD1/Co-REST/HDAC1 complex. This occurs through binding PRC2 and the LSD1/Co-REST/HDAC1 complex at 5’ and 3’ ends of HOTAIR respectively (70). This 3’ bound complex is stabilized by the Zinc Finger protein 198 (ZNF198) and is responsible for the removal of gene activating histone acetylations, and H3K4 methylations (71). The RNA helicase DEAD-box helicase 5 (DDX5) also associates with PRC2 *via* the HOTAIR scaffold and is essential for maintaining the stability of its SUZ12 component (15). The coupling action of HOTAIR therefore reinforces gene repression by these two chromatin-modifying complexes.

During HBV infection, the cccDNA minichromosome is known to associate closely with HOTAIR-encoding chromatin, although the significance of this interaction is not quite clear (72). The HBV minichromosome assumes a chromatin-like state which is also subject to epigenetic silencing by PRC2 thus preventing viral replication (15). This repression is alleviated by miRNA-mediated silencing of the RNA helicase DDX5 (73). Loss of DDX5 subsequently leads to the destabilization of SUZ12 (15). HBx also activates polo-like kinase1 (PLK1), which then phosphorylates both SUZ12 and ZNF128 marking them for ubiquitination by the E3 ligase Mex3b (14, 15, 74). In this scenario, the HOTAIR scaffold is hijacked by Mex3b to facilitate ubiquitination of SUZ12 and ZNF198, targeting them for proteasomal degradation. Resulting destabilization of PRC2 leads to de-repression of viral chromatin and initiation of viral replication. PRC2-repressed genes, such as *EpCAM* and pluripotency genes (*Nanog, Sox2* and *Oct4*), which are commonly expressed in progenitor hepatic cancer cells, are also upregulated through this mechanism, and are indicative of poor prognosis following tumor resection (15, 60).

In addition to relieving epigenetic repression of cccDNA, HOTAIR actively enhances HBV promoter functions. It does so by guiding the transcription factor SP1 to viral gene promoter sites to upregulate viral transcription and replication (16).

#### 3.1.8 HULC

Highly upregulated in liver cancer (HULC) is another lincRNA that is progressively upregulated in HBV-related disease states (57). This polyadenylated 500 nucleotide transcript was originally found to be upregulated in HCC tissues and is mainly localized in the cytoplasm. However, it also plays vital roles in the nucleus (75). Transcription of HULC falls under the control of a CREB transcription factor-dependent promoter and since HBx can interact with and enhance the affinity of CREB to its promoter, HULC can be indirectly upregulated by HBx (18, 76).

Upregulation of HULC is important to stabilize cccDNA and promote HBV replication (40). Two antiviral cytidine deaminases, APOBEC3A and APOBEC3B, are essential for the degradation of cccDNA and prevention of persistent infections (77). HULC prevents this degradation by upregulating expression of miR-539, which post-transcriptionally silences APOBEC3B. This transcriptional activation of miR-539 is not directly mediated by HULC. Instead, HULC stimulates HBx to activate and recruit the transcription factor STAT3 to the miR-539 promoter.

Another nuclear target of HULC is the downstream gene encoding the tumor suppressor protein p18 (18). In response to DNA damage, p18 is translocated to the nucleus to activate p53 and halt cell-cycle progression (78). However, in HCC tissues high levels of HULC were associated with low levels of p18. It was subsequently shown that HULC downregulates p18 by interacting with its promoter to repress transcription, leading to increased cell proliferation. Thus, in addition to promoting HBV persistence HULC also directly contributes to development of HCC by silencing the tumor suppressor p18.

#### 3.1.9 MALAT1

Metastasis-associated Lung Adenocarcinoma Transcript 1 (MALAT1) is a multi-functional lincRNA that is upregulated in HCC tissues and even more so in HBx-expressing hepatocytes (79). It is initially transcribed as a single lncRNA but is cleaved into two separate molecules that are destined for different cellular compartments (80). The larger non-coding RNA is retained in the nucleus where it functions in a number of ways to promote various types of malignancies, whereas the smaller non-coding RNA is shuttled to the cytoplasm. Both RNAs can activate the ERK/MAPK pathway to promote metastasis in HCC, although the exact mechanism is yet to be elucidated (81).

In the nucleus, MALAT1 also functions as an enhancer-like lncRNA by recruiting the transcription factor Sp1 to the promoter of the *Latent Transforming Growth Factor β-Binding Protein 3 (LTBP3)* gene, which is closely located on the same chromosome, but transcribed in the opposite orientation (20). MALAT1 binds to both Sp1 and the LTBP3 promoter forming an RNA-protein-DNA triplex which guides the transcription factor to and stabilizes its interaction with the Sp1 consensus sequence located on the LTBP3 promoter. LTBP3 is a glycoprotein that is secreted into the extracellular matrix and promotes the proper folding and secretion of TGF-β (82). LTBP3 also enhances metastatic potential in several cancer cell lines and animal malignancy models (83). In HBx-expressing hepatocytes, upregulation of LTBP3 results in an increase in N-cadherin and vimentin, and a decrease in E-cadherin, which increases the invasive and migratory properties of hepatocytes *in vitro* and *in vivo* (79). This effect is mitigated by the knockdown of MALAT1, thus the HBx/MALAT1/LTBP3 axis could be considered a therapeutic target to prevent EMT.

#### 3.1.10 Linc01152

Linc01152 was initially identified as a downregulated lncRNA in HBV-related HCC tumor tissues and was thought to be a tumor suppressor (47). However, a later study showed that the lncRNA is upregulated in HBx-expressing cell lines and can promote cell proliferation *in vitro* and *in vivo* (22). In HBV-related HCC patients, tissue and serum levels of IL-23 were positively correlated with levels of linc01152. *In vitro* experiments revealed that linc01152 binds directly to the IL-23 promoter and activates transcription of this pro-inflammatory cytokine. In a separate study, IL-23 levels correlated with HBV DNA levels, and were shown to mediate liver damage by expanding Th17 cells (21). Overexpression of linc01152 was associated with an upregulation of STAT3 and phosphorylated STAT3, whereas silencing of STAT3 reduced cell proliferation (22). Thus, linc01152 may promote HCC by activating the STAT3 pathway.

### 3.2 Alternative Splicing

Alternative splicing occurs in the nucleus and provides a means of producing different mature mRNAs from a single precursor mRNA. Transcriptome comparisons between HCC and normal hepatic tissues have shown differential alternative splicing patterns of certain precursor mRNAs, which gives rise to novel protein isoforms that promote tumorigenesis [reviewed in (84)]. LncRNAs are also subject to alternative splicing, but some have also demonstrated the ability to alter splicing patterns by interacting with splicing factors or precursor mRNAs [reviewed in (85)].

One such lncRNA, the Zinc finger E-box binding Homeobox 2 antisense RNA1 (ZEB2-AS1), may promote HBV-related HCC by this mechanism. ZEB2-AS1 overlaps the donor splice site of the *ZEB2* gene (86). ZEB2 is a transcriptional repressor of E-cadherin and is thus a mediator of EMT (87). The mRNA that encodes ZEB2 has an unusually long 5’ UTR which contains an internal ribosome entry site (IRES) that is necessary for translation. In normal epithelial cells, this mRNA undergoes splicing to remove a major portion of its 5’ UTR including the IRES, leaving behind a shorter 5’ UTR which contains a sequence that inhibits translation. Thus, ZEB2 mRNA cannot be translated, and the cell maintains an epithelial phenotype.

**Figure 3:**
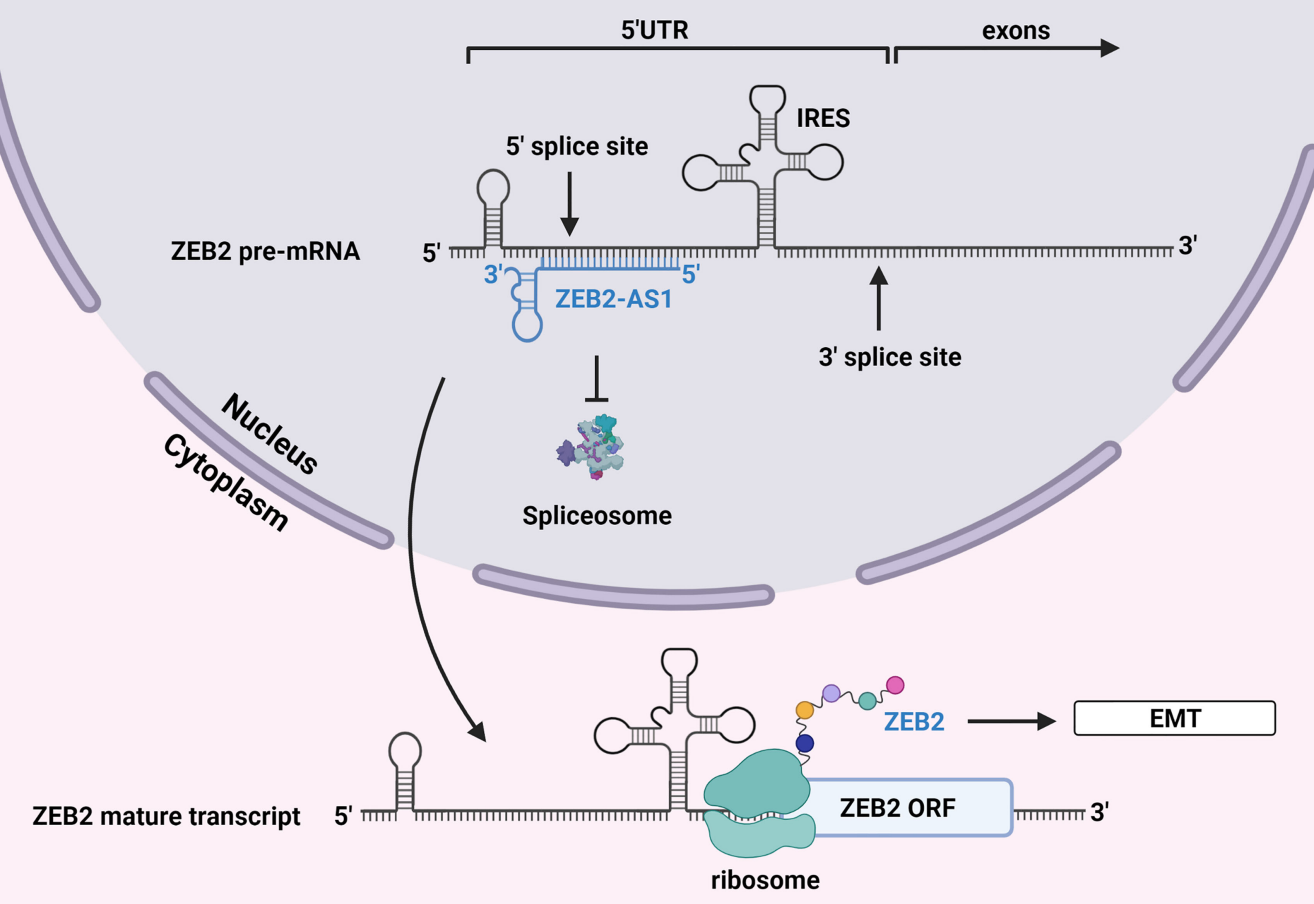
Alternative splicing by lncRNA ZEB2-AS1. The antisense lncRNA ZEB2-AS1 binds to the 5’ UTR donor splice site of mRNA encoding ZEB2, a transcriptional repressor of E-cadherin. This prevents the spliceosome from eliminating this region of the transcript, which contains an IRES that is necessary for translation. ZEB2 is subsequently translated, and its increased levels promote EMT. Created with BioRender.com.

In the case of HBV-related HCC, increased levels of HBx were associated with increased levels of ZEB2-AS1 and the transition into a mesenchymal phenotype (23). Being an antisense lncRNA, ZEB2-AS1 forms an RNA-RNA duplex through complementary base pairing with the 5’ splice site of the precursor mRNA encoding ZEB2 (86). This prevents binding of the spliceosome, and the longer IRES-containing 5’ UTR is retained in the mature transcript allowing for ZEB2 to be translated. Increased levels of ZEB2 then suppresses transcription of E-cadherin and promotes EMT (**Figure 3**).

### 3.3 Competitive Endogenous RNAs (ceRNAs)

**Figure 4:**
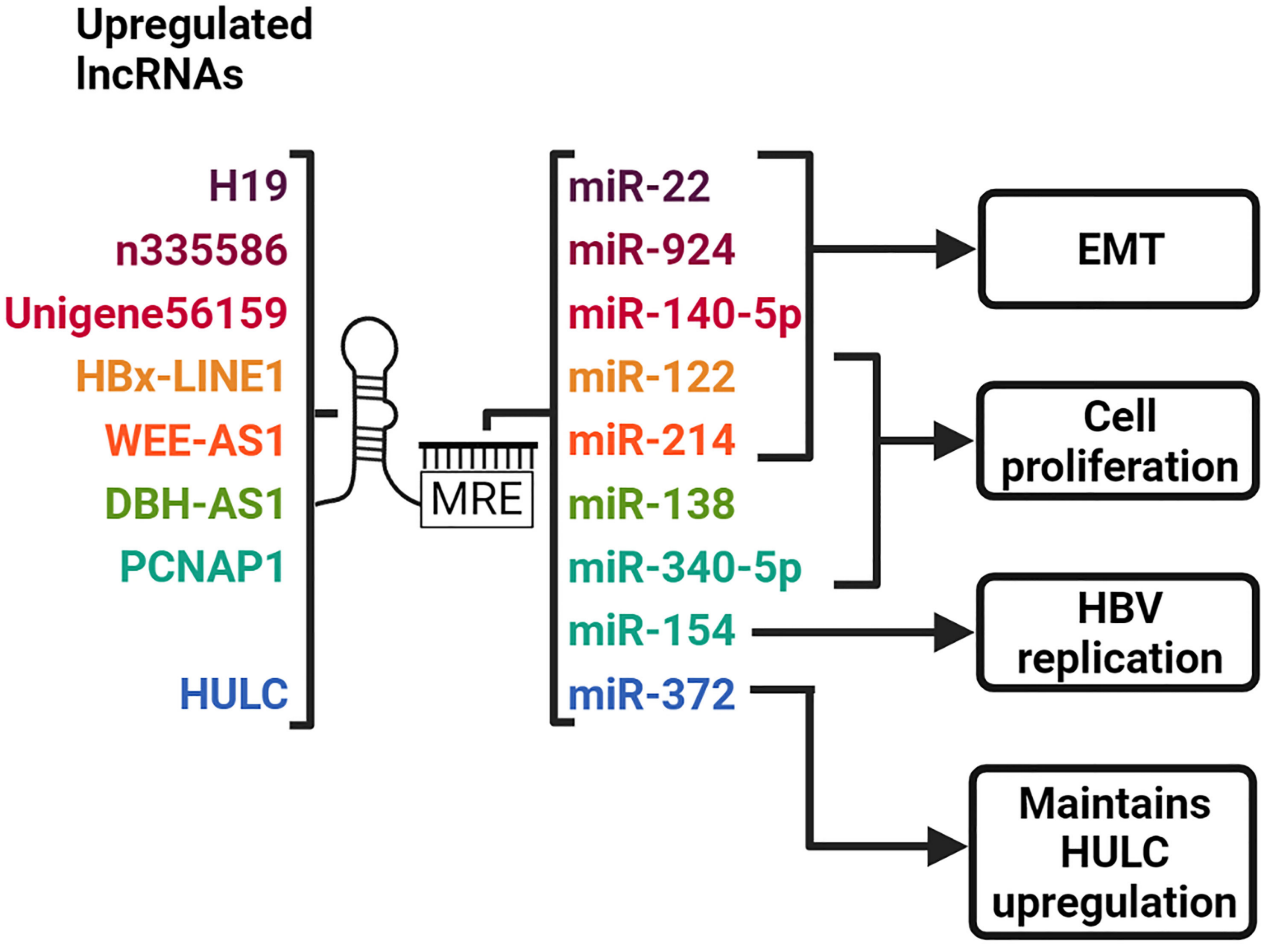
LncRNAs act as ceRNAs to sequester complementary miRNAs. LncRNAs harbor MREs which allow them to bind to miRNAs with complementary sequences. H19, n335586 and Unigene56159 promotes EMT by sequestering miR-22, miR-924 and miR-140-5p, respectively. HBx-LINE1 and WEE2-AS1 promotes EMT and cell proliferation by sponging miR-122 and miR-214, respectively. DBH-AS1 increases cell proliferation by sequestering miR-138. PCNAP1 sponges miR-340-5p and miR-154 to promote cell proliferation and HBV replication, respectively. HULC sequesters miR-372, thereby maintaining its upregulation. Created with BioRender.com.

ceRNAs are found in the cytoplasm and mediate post-transcriptional gene regulation. This type of lncRNA contains inherent miRNA response elements (MREs), allowing them to sequester or sponge complementary miRNAs (**Figure 4**). This action reduces availability of key miRNAs with tumor suppressor functions, thus facilitating de-repression of their oncogenic target molecules [reviewed in (88)].

#### 3.3.1 HBx-LINE1

HBx-LINE1 is a unique chimeric lncRNA composed of the *HBx* and human LINE1 transcripts. It is formed by the integration of HBV DNA into the host *LINE1* gene and activates the *LINE1* sequence (24). When investigated in HBV-positive HCC cell lines, HBx-LINE1 was actively transcribed by the HBx promoter, from the site of viral integration on chr.8p11.21. HBx-LINE1 expression was also observed in 23.3% to 42.5% of HBV-positive HCC tumors, and correlated with reduced overall survival (24, 25). Furthermore, *in vitro* investigations revealed that HBx-LINE1 enhanced migration and invasion by inducing EMT, and promoted the translocation of β-catenin into the nucleus where it activates the Wnt signaling pathway.

In HBx-LINE1-positive HCC tissues and cells, the HBx-LINE1 transcript was shown to contain six confirmed binding sites for the tumor suppressor miR-122 (25). Specific binding of miR-122 to each of the corresponding HBx-LINE1 sites, and the inverse correlation between these transcripts, suggests that HBx-LINE1 acts as a molecular sponge that sequesters miR-122. Reduced levels of miR-122 in HBx-LINE1-positive HCC cells were associated with reduced E-cadherin and increased WNT1, phosphorylated β-catenin, migration, invasion and proliferation. Overexpression of miR-122 in these cells reversed these effects and significantly depleted HBx-LINE1 expression. When assessed in the mouse liver, HBx-LINE1 was found to sequester miR-122, which led to reduction of E-cadherin, promotion of β-catenin signaling and initiation of abnormal mitosis and liver injury. The evidence from these studies suggests that HBx-LINE1 sequesters miR-122 which leads to tumor progression by the induction of EMT and consequent activation of the Wnt/β-catenin signaling pathway (24, 25).

#### 3.3.2 DBH-AS1

The HBx-induced expression of the lncRNA DBH-AS1 in HBV-related HCC is thought to involve the tumor suppressor protein p53. Studies have revealed a putative p53 binding site upstream of DBH-AS1 and have also shown that p53 suppression results in increased expression of DBS-AS1 (26). Interestingly, HBx is known to inhibit p53 to promote HCC (89). It is thus likely that HBx inhibition of p53 relieves transcriptional repression of DBH-AS1. Overexpression of DBH-AS1 in HBV-related HCC has been associated with decreased apoptosis and cell proliferation and is mediated by the activation of the ERK/p38/JNK/MAPK pathway, although the exact mechanism by which this occurs has not yet been elucidated. Activation of this pathway downregulates the cyclin-dependent kinase inhibitors p16, p21, and p27. The downstream effects of this suppression include the upregulation of the cyclin-dependent kinase CDK6, and cyclins D1 and E1, which promote G1/S and G2/M cell cycle progression with cell proliferation. A subsequent study uncovered that DBH-AS1 functions as a molecular sponge for miR-138, which leads to activation of the FAK/Src/ERK pathway and results in HCC (27). A correlation between DBH-AS1 and HBsAg levels in infected individuals and tumor size in HBV-induced HCC has been shown (26).

#### 3.3.3 PCNAP1

The lncRNA PCNAP1 is a pseudogene of *PCNA* (90). PCNAP1 is highly expressed in HBV-positive HCC cells and patient tumor tissues (28, 29). Its upregulation was also observed in the livers of HBV-infected chimeric mice harboring human hepatocytes (28). This suggests that PCNAP1 expression is positively associated with HBV infection and HBV-related HCC. PCNAP1 has more than one target miRNA binding site. In the study performed by Feng et al, the results indicated that PCNAP1 acted as a ceRNA to sponge the tumor suppressor miR-154, thereby preventing it from inhibiting PCNA expression. This allows PCNA to bind cccDNA through interaction with the HBV core protein, promoting HBV replication and cccDNA accumulation. In the more recent study by He et al., PCNAP1 was suggested to promote HCC cell proliferation by sequestering the tumor suppressor miR-340-5p, and preventing it from directly suppressing ATF7 expression in HCC cells (29). Furthermore, PCNAP1 overexpression was significantly associated with reduced overall survival and may be a potential predictor of HCC prognosis.

#### 3.3.4 WEE2-AS1

The antisense lncRNA WEE2-AS1 is upregulated in HBx-expressing HCC cell lines and tissues (30). It is associated with multiple hallmarks of HCC including increased cell proliferation, migration, invasion, hepatic vascular invasion, and decreased apoptosis (30). The Fermitin family member 3 (FERMT3) was identified as the downstream target of WEE2-AS1. Deregulation of FERMT3 is observed in various types of cancers, including HBV-related HCC, and involves activation of the Akt signaling pathway. The exact mechanism by which FERMT3 is upregulated by WEE2-AS1 is not yet known. However, molecular interaction prediction tools identified both WEE2-AS1- and FERMT3-encoding mRNA as targets for miR-214. In the case of glioblastoma, WEE2-AS1 is enriched in the cytoplasm and acts as a molecular sponge for other miRNAs, thus it is possible that it may act as a molecular sponge to sequester miR-214 and inhibit post-transcriptional silencing of FERMT3 (31).

#### 3.3.5 Unigene56159

The lncRNA Unigene56159, of approximately 2,653 nucleotides in length, is located in the second intron of the *ROBO1* gene (32). Its expression is significantly upregulated in HBV-related HCC tissues and cells, and it promotes cell migration, invasion, and EMT *in vitro*. Further investigation revealed that Unigene56159 may function as a ceRNA by acting as a sponge to sequester miR-140-5p and inhibit repressive activity on its target Slug, which is a repressor of the adhesion molecule E-cadherin (32, 33). The sequestration of miR-140-5p by Unigene56159 therefore promotes EMT which leads to hepatoma cell migration and invasion.

#### 3.3.6 H19

H19 was one of the first lncRNAs to be discovered (91). It is approximately 2,300 nucleotides long and is an oncofetal lncRNA that is transcribed only from the maternal allele of the *H19* gene during embryogenesis, and is suppressed postnatally (92). H19 expression is regulated by c-Myc which binds and promotes histone acetylation of E-boxes within the H19 promoter leading to initiation of transcription (93). Over the years, multiple studies have revealed that expression of this lncRNA is resumed in several cancers, where it exhibits either tumor suppressor or oncogenic properties [reviewed in (94)]. In HCC, the role of H19 is somewhat controversial and requires further examination. In the tumor tissue of patients with HBV-related HCC, H19 is significantly upregulated and is positively associated with lymph node invasion and distal metastasis, and negatively correlated with overall patient survival (95). Knockdown of H19 in HepG2.2.15 cells inhibited cell proliferation, migration, and invasion. This was accompanied by reduced expression of N-cadherin, Vimentin, β-catenin, and Matrix Metalloproteinase-9, all of which promote EMT. Using online prediction tools, miR-22 was identified as a putative target of H19 and was subsequently shown to be negatively regulated by H19 in HCC tissues and cell lines. The inhibition of migration and invasion induced by H19 knockdown was also rescued by the knockdown of miR-22. Thus, these results suggest that H19 promotes progression of HBV-related HCC by sponging miR-22 to increase the expression of EMT-associated proteins (95). Interestingly, H19 promoter-driven expression of the a diphtheria toxin has been evaluated as treatment for cancer [reviewed in (96)]. Since H19 is abundantly expressed in certain tumors and not surrounding tissues, delivery of a diphtheria toxin expressing plasmid under the transcriptional control of the H19 promoter has the potential to kill cancerous cells selectively.

In direct contrast to its role as a promoter of EMT in HBV-related HCC, H19 inhibited EMT in HCC in an earlier study (36). H19 expression was reduced in HCC tumor tissues compared to the adjacent peritumoral tissues, and lower H19 tumor/peritumoral ratios were associated with intrahepatic tumor metastasis, reduced tumor capsule integrity, and decreased disease-free survival. H19 expression was also reduced in invasive HCC specimens compared to non-invasive HCC specimens suggesting its importance in suppressing EMT. Investigation of HCC cell lines *in vitro*, revealed that H19 may reverse EMT by enhancing the expression of the EMT suppressor miR-200 family. This increase in miR-200 expression is brought about epigenetically through recruitment of the histone acetylase HnRNP U/PCAF/RNA PolII protein complex to chromatin regions upstream of the miR-200 family.

#### 3.3.7 HULC

In addition to transcriptional silencing of p18, HULC acts as a molecular sponge for multiple cytoplasmic miRNAs. One of these targets is miR-372 which mediates translational suppression of a cAMP-dependent protein kinase, PRKACB (19). De-repression of PRKACB mRNA by miR-372 sponging increases the amount of PRKACB protein available to phosphorylate and activate CREB, which as mentioned earlier, is required for upregulating expression of HULC. Thus, HULC is part of an autoregulatory loop that serves to maintain its upregulation in HBV-related HCC.

#### 3.3.8 n335586

LncRNA n335586 is located within the *CKMT1A* gene and has a length of approximately 270 nucleotides (37). This lncRNA is highly expressed in HBV-positive HCC tissues and cells. This is associated with decreased expression of E-cadherin and cytokeratin, and increased expression of vimentin pointing to a role in EMT. LncRNA n335586 functions in *cis* by upregulating expression of its host gene *CKMT1A* which was associated with increased migration and invasion of HCC cells. This finding, and the fact that n335586 overlaps with the downstream portion of the CDS and the 3’ UTR of the *CKMT1A* gene, suggests that n335586 may function as a ceRNA to prevent miRNA-mediated degradation of CKMT1A. The tumor suppressor miR-924 bound n335586 and CKMT1A transcripts competitively, which led to enhanced migration, invasion, and EMT *in vitro* and metastasis *in vivo*. Thus, n335586 promotes EMT by acting as a molecular sponge for miR-924 which alleviates post-transcriptional repression of the CKMT1A.

### 3.4 Protein Interactions

**Figure 5:**
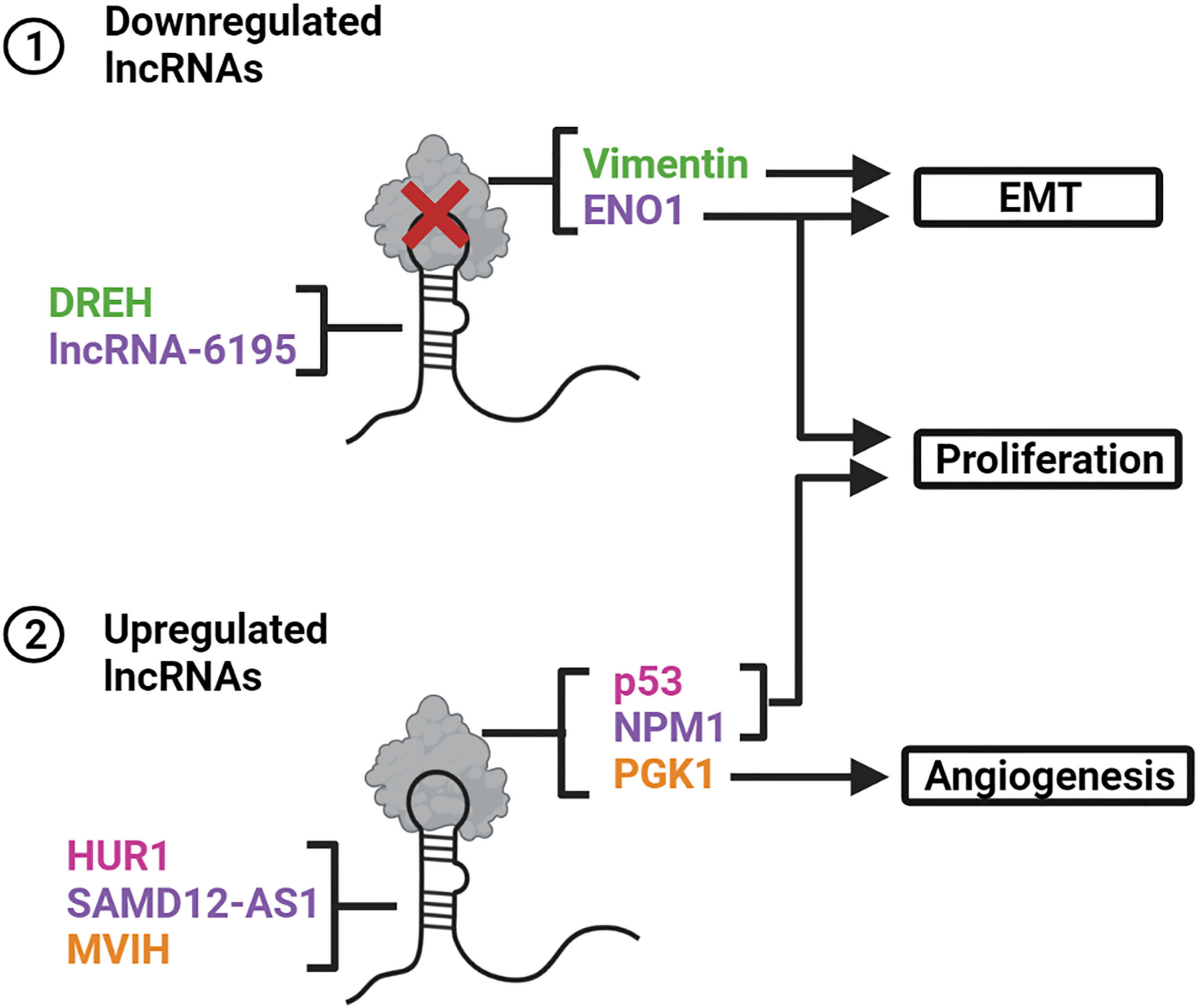
LncRNAs interact with proteins and alter their stability or functionality. **(1)** DREH and LncRNA-6195 are downregulated in HBV-related HCCs. The lack of interaction between DREH and the protein vimentin promotes EMT. Deficient binding of LncRNA-6195 to ENO1 promotes EMT and cell proliferation. **(2)** HUR1, SAMD12-AS1 and MVIH are upregulated in HBV-related HCCs. HUR1 and SAMD12-AS1 enhance cell proliferation by inhibiting p53 and NPM1, respectively. MVIH interacts with PGK1 to prevent its secretion thereby promoting angiogenesis. Created with BioRender.com.

Apart from interacting with proteins that make up chromatin-modifying complexes in the nucleus, certain lncRNAs can post-translationally regulate oncogenic or tumor suppressor proteins found in both the nuclear and cytoplasmic compartments. They enhance protein stability by either binding directly to the target protein or by preventing ubiquitination and proteasomal degradation [reviewed in (51)]. Conversely, binding of lncRNAs can also inhibit protein functionality or promote protein degradation (**Figure 5**).

#### 3.4.1 DREH

LncRNA DREH was originally discovered in mice. Its expression is inhibited by HBx, as demonstrated by its significant downregulation in the livers of HBx-transgenic mice and HBx-expressing mouse liver cells (38). Suppression of DREH in mouse liver cells *in vitro* leads to increased cell proliferation, migration, and invasion, whereas *in vivo* investigations revealed DREH overexpression represses tumor growth and metastasis. DREH interacts with and inhibits the intermediate filament protein vimentin. Further analysis in murine hepatoma cells revealed that DREH may cause the cytoskeleton to reverse from a high to a low migration phenotype by altering the filamentous structure of vimentin. These results suggests that DREH acts as a tumor suppressor by interacting with the vimentin protein and reducing its expression, thereby altering the cytoskeleton structure to inhibit tumor metastasis.

Sequence analysis revealed *hDREH*, located on human chromosome 5, as a possible human ortholog of the murine *DREH* (38). The lncRNA hDREH transcribed from this locus was significantly downregulated in HBV-related HCC tissues. Its downregulation was associated with reduced recurrence-free survival, reduced overall survival, enhanced tumor size and increased HBsAg (38, 39). hDREH expression was inversely associated with HBx concentration in human HBV-related HCC tissues and significantly downregulated in HBx-expressing human hepatoma cells (39). Subsequent investigations revealed that hDREH inhibition of expression enhances cell proliferation *in vitro* and tumor growth *in vivo*. These observations further support a role for hDREH as a tumor suppressor in HBV-related HCC.

#### 3.4.2 SAMD12-AS1

SAMD12-AS1 is an antisense lncRNA that is approximately 700 nucleotides long and shares the same locus as *SAMD12* (40). This lncRNA is upregulated in HBV-positive HCC tissues and HBx-expressing cells and its transcription is enhanced by HBx. SAMD12-AS1 enhances proliferation and inhibits apoptosis, as indicated by its effect to reduce both caspase-3/7 activity and PARP-1 cleavage. The transcript primarily interacts with the nucleophosmin (NPM1) protein in the nucleus. NPM1 interacts with the E3 ubiquitin ligase HDM2 and prevents the degradation of p53 (97). Subsequent investigations revealed that p53 was rapidly degraded in SAMD12-AS1-expressing cells through increased ubiquitination of p53 by HDM2 (40). These results demonstrate that SAMD12-AS1 competitively binds to NPM1 to prevent its interaction with HDM2. This increases availability of HDM2 to mark p53 for proteasomal degradation, which ultimately promotes cell proliferation and inhibits apoptosis.

#### 3.4.3 HUR1

The *HUR1* gene transcribes the lncRNA HUR1, which is approximately 1,200 nucleotides in length (41). HUR1 upregulation was positively associated with HBV viral load in patients with HBV-related HCC. Its increased expression was also observed in HBx-expressing HepG2 cells, which indicated that HUR1 is upregulated by HBx. In HUR1-expressing HepG2 cells, HUR1 enhanced proliferation, as evidenced by the increased PCNA expression, and promoted G1/S transition. Enhanced proliferation was also observed in nude mice that were injected with these cells. Additional *in vitro* studies revealed that HUR1 enhances proliferation by interacting with the tumor suppressor p53 and inhibiting its transcriptional regulation of p21 and Bax. Furthermore, HUR1 promoted cell proliferation and liver regeneration in HUR1 transgenic mice that underwent partial hepatectomy, and promoted tumor progression in DEN-induced HCC mouse models.

#### 3.4.4 MVIH

LncRNA associated with microvascular invasion in HCC (MVIH) is found within the intron of the *RPS24* gene, however MVIH and *RPS24* are independently transcribed (42). This lncRNA is highly upregulated in HBV-related HCC and is associated with greater microvascular invasion and advanced tumor node metastasis T stage. It is also an independent predictor of recurrence-free survival after hepatectomy. When examined *in vivo*, MVIH overexpression promoted tumor growth and intrahepatic metastasis. MVIH interacts directly with the angiogenesis inhibitor phosphoglycerate kinase (PGK1) and that there is a negative correlation between the two. This negative association was also observed in patient samples. Patients with greater MVIH expression in their tumor tissues showed reduced PGK1 serum levels. These results indicate that MVIH induces tumor angiogenesis by inhibiting the secretion of PGK1, thus promoting tumor growth and intrahepatic metastasis.

#### 3.4.5 LncRNA-6195

LncRNA-6195 is significantly downregulated in HBV-related HCC tissues from patients (43). Its downregulation was correlated with HBx and decreased overall survival. LncRNA-6195 inhibited tumor growth *in vitro* and *in vivo*. RNA immunoprecipitation analysis revealed that lncRNA-6195 bound the substrate-binding site of ENO1 (aa 237-405). In lncRNA-6195-expressing hepatoma cells, lncRNA-6195 had no effect on ENO1 expression, but reduced ENO1 enzyme activity (43). The results demonstrate that lncRNA-6195 is a tumor suppressor which inhibits tumor growth by binding to the substrate region of ENO1. Resultant inhibition of enzyme activity represses energy metabolism in hepatoma cells.

### 3.5 miRNA Precursors

**Figure 6:**
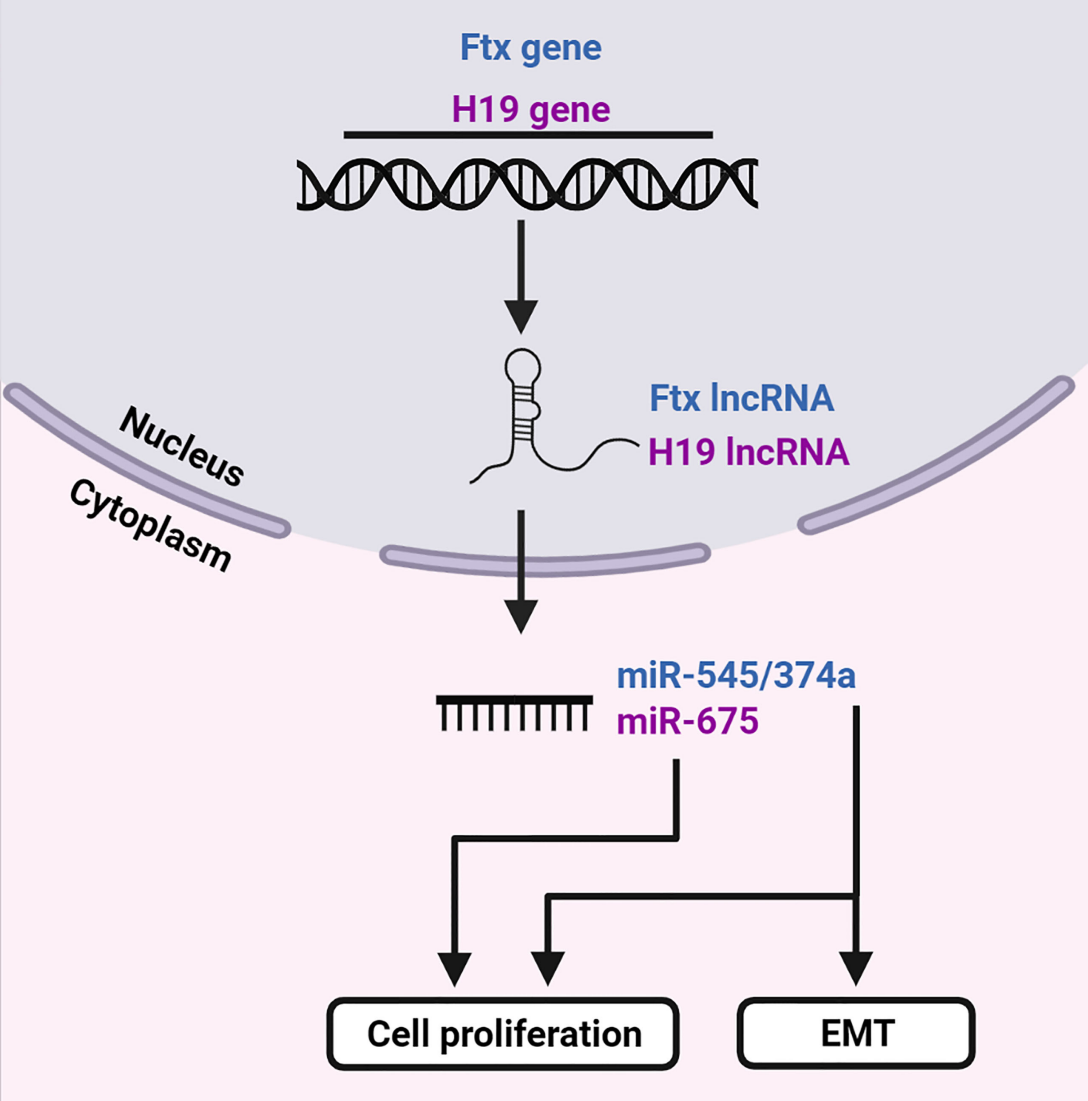
LncRNAs are precursors of miRNAs. The *Ftx* and *H19* genes transcribe lncRNAs Ftx and H19, respectively. LncRNA Ftx encodes the miR-545/374a cluster, which subsequently promotes EMT and cell proliferation. LncRNA H19 transcribes miR-675 which enhances cell proliferation. Created with BioRender.com.

A lesser employed mechanism of post-transcriptional gene regulation is mRNA suppression by miRNA-encoding lncRNAs. These lncRNAs are transcribed as precursors of miRNAs and contain clusters of miRNAs (**Figure 6**). Subsequent downregulation of their target mRNAs may promote development of HCC.

#### 3.5.1 Ftx

The *Ftx* gene is located within the X-inactivation center of the X chromosome (98). It transcribes a 2,300 nucleotide long highly conserved lncRNA, which encodes four miRNAs in its introns. The miR-545/374a and miR-421/374b clusters are encoded by intron b and intron 12, respectively [reviewed in (99)]. In a study performed by Zhao et al., the miR-545/374a cluster was highly expressed in HBV-related HCC and was positively associated with histological differentiation and incomplete tumor capsule formation, whereas the upregulation of miR-374a was observed in patients with distal metastasis (45). When investigated *in vitro*, miR-545 and miR-374a promoted cell proliferation, migration and invasion which suggests a possible role in tumorigenesis. In HBV-related HCC patient tissue samples, expression of HBx correlated significantly with miR-545. In patients that were positive for HBeAg, miR-374a was highly expressed, whereas in patients that were positive for HBV DNA, both miRNAs were expressed. Furthermore, miR-545 was greatly increased in the HBV-positive cell lines and both miRNAs were significantly increased in cells transfected with HBV DNA. This positive association between the miR-545/miR-374a and HBV infection indicates that its expression may be dependent on HBx. Zhao et al. then revealed that expression of miR-545 and miR-374a in the HBV-related HCC tissues correlated with concentrations of the miRNAs in patient serum samples. After tumor resection, these concentrations decreased significantly, suggesting that the miRNAs are tumor-derived. Levels of miR-545 and miR-374a were also increased in the serum of patients with alpha-fetoprotein levels ≥20 ng/ml. Therefore, the detection of these miRNAs in the serum of patients infected with HBV may be useful as a marker of HCC. The levels of miR-545/374a were also significantly higher in HBV-related HCC from males compared to females (45). Low concentrations in females was hypothesized to be related to the estrogen-related gene ESRRG, which is a potential target of miR-545. In a study performed by Liu et al., where the majority of the patients with HCC were positive for HBsAg, Ftx and miR-545 were significantly upregulated in patient tumor tissues (44). This was associated with tumor size, high histological grade, advanced tumor stage, shorter overall survival and decreased disease-free survival, whereas increased expression of miR-545 alone was associated with venous infiltration.

#### 3.5.2 H19

In addition to its previously mentioned functions, H19 was also identified as a precursor of miR-675 (100). The expression of H19 and miR-675 was significantly upregulated in the tissues of patients with chronic HBV and HBx-transfected LO2 cells. Inhibition of either of these transcripts within the HBx-transfected LO2 cells enhanced cell viability, suppressed HBx-induced apoptosis, inflammatory cytokine production, oxidative stress, and modulated energy metabolism. However, these effects were partially reversed by knocking down PPARα (40). PPARα was identified as a direct target of miR-675 and was downregulated in the tissue of patients with chronic HBV. PPARα expression was reduced by overexpression of HBx in LO2 cells. This suppression was (partially) reversed by inhibition of H19 or miR-675. These findings suggest that the overexpression of HBx induces H19 and miR-675 upregulation, leading to the inhibition of PPARα, which then promotes Akt/mTOR signaling.

### 3.6 LncRNAs With Unknown Mechanisms in HBV-Related HCC

The mechanisms of action of many lncRNAs dysregulated in HBV-related HCC remain largely unknown. Some have had their functions examined in other types of cancers. However, these lncRNAs may elicit other effects in HBV-related HCC. The proposed functions and targets of recently discovered lncRNAs have been predicted using *in silico* tools. A few of these lncRNAs are mentioned below.

The lncRNA TUC338 is upregulated in HBV-infected primary human hepatocytes as well as HCC cell lines and tissues, and has been shown to modulate expression of regulatory proteins involved in cell-cycle progression (12, 101). TUC338 associates with chromatin at multiple DNA motifs, the most enriched being those that are homologous to the binding sites of the tumor suppressors p53 and Pax6 (102). Thus, upregulation of TUC338 could potentially competitively inhibit p53 and Pax6, and subsequently suppress their downstream target genes. TUC338 also post-transcriptionally stabilizes mRNA encoding the Plasminogen Activator Inhibitor (PAI) protein, which is upregulated in HCC and contributes to transformed cell growth. This interaction is not sequence-specific, but rather mediated by the PAI-1 RNA binding protein (PAI-RBP1) which is also positively regulated by TUC338. This mechanism of tumorigenesis has not yet been confirmed in HBV-related HCC.

As with many aforementioned lincRNAs (such as HEIH, UCA1 and linc00152), ULK4P2 associates with EZH2, but the exact gene target of ULK4P2 has not been elucidated (103). Analysis of a ULK4P2-mRNA co-expression network has pointed to its potential involvement in cell proliferation and metastasis. It is possible that ULK4P2 may mediate changes to chromatin architecture to enable transcription of genes involved in promoting cell proliferation and EMT. ANRIL was initially not correlated with HBV in HCC tissues (104, 105). However, this was contradicted in a more recent study, as ANRIL expression was significantly increased in patients with HBV-related HCC (15). The discrepancy may be attributed to differences in sample size between studies. The former compared 103 and 67 HBV-positive patients against 34 and 10 negative cases, while the latter compared 11 HBV-positive HCC cases against 52 negative cases.

LncRNAs PRC1-AS1, LINC00665 and AC092171.4 are highly upregulated in HBV-infected HCC cell lines (106). AF085935 and uc003wbd are significantly expressed in the serum of HBV-infected HCC patients suggesting utility as potential biomarkers for detection of the disease (107). LncRNAs AX800134 and uc001ncr are significantly upregulated in the serum and tumor tissue of patients with HBV-related HCC, including those with alpha-fetoprotein <400 ng/ml or early-stage disease (BCLC 0+A) (108, 109). Thus, these lncRNAs may be potential biomarkers for early diagnosis of the disease. AX800134 was upregulated in HepG2 cells which express HBx (109). Elevated levels of the pro-inflammatory cytokine TNF-α induced NF-κB signaling, which upregulated AX800134 in these cells. This upregulation was reversed by the reactive oxygen species scavenger, PDTC. Silencing of AX800134 did not decrease expression of the HBx protein but significantly inhibited cell growth rate and invasion, and enhanced apoptosis in serum-starved cells. This suggests that the presence of HBx and chronic inflammation may induce expression of AX800134, which in turn promotes hepatoma cell growth and invasion.

The Gene Expression Omnibus database was used to identify BAIAP2-AS1 as a lncRNA that was significantly upregulated in HBV-related HCC (110). Gene set enrichment analysis showed that transcripts encoding MAPKAP1, RAF1 and E2F3 were enriched with BAIAP2-AS1 expression. *In silico* sequence analysis of BAIAP2-AS1 identified binding sites for miR-491, miR-331 and miR-34A. These miRNAs target MAPKAP1, RAF1 and E2F3 respectively. Knockdown of BAIAP2-AS1 in HepG2 cells decreased MAPKAP1 and RAF1 expression, which suggests that BAIAP2-AS1 may function as a ceRNA.

RNA-sequencing data from The Cancer Genome Atlas (TGCA) database and microarray datasets from the European Bioinformatics Institute database revealed eight lncRNAs (TSPEARAS1, LINC00511, LINC01136, MKLN1-AS, LINC00506, KRTAP5-AS1, ZNF252P-AS1 and THUMPD3-AS1) that may be a collective prognostic biomarker for HBV-infected patients with HCC, as they were significantly correlated with overall survival (111). Further investigation into the mechanisms of these lncRNAs revealed that they may be involved in tumorigenesis by regulating cell cycle-related processes or pathways. Similarly, in another study which analyzed lncRNA expression using bioinformatics, seven lncRNAs (PVT1, LINC01138, LINC02499, AL355488.2, FGF14-AS2, MAFG-AS1 and LINC00261) were significantly associated with overall survival and collectively were identified as a prognostic signature for patients with HBV-related HCC (112).

MSC-AS1, POLR2J4, EIF3J-AS1, SERHL, RMST, and PVT1 were proposed to be a six-lncRNA prediction marker for HBV-HCC recurrence-free survival. This was determined by univariate COX regression analysis and validated in the TCGA database (113). Another study which utilized bioinformatics analysis revealed that FAM182B-miR-125b-5p-E2F2 and LINC00346-miR-10a-5p-CDK1/CCNE1 ceRNA axes enhanced tumorigenesis by promoting HBV-related HCC cell growth, thus making these lncRNAs attractive therapeutic targets and potential disease predictors (95). Deep sequencing analysis identified significant downregulation of n346077 expression in HBV-positive HCC tissues. Further *in vitro* investigations in HepG2 and QGY-7703 cell lines revealed that n346077 may act as a tumor suppressor by inhibiting hepatoma cell invasion and migration (114).

## 4 Conclusion

Over the past few years, there has been an unprecedented increase in the identification of dysregulated lncRNAs in HBV-related HCC. These lncRNAs are quite heterogenous in size, type, structure, cellular location, and functionality. Through an intricate network of interactions, involving epigenetic modifications to chromatin, modulation of transcription factors, sponging of miRNAs, protein interactions and miRNA production, these lncRNAs work in concert to promote tumorigenesis and cancer progression, while also modulating HBV replication. Some of these lncRNAs are common to many other types of cancers, however, the manner in which an individual lncRNA functions sometimes varies across cancers. Thus, the mechanisms described in this review are limited to those which have been confirmed in HBV-related HCC. Other reported mechanisms of lncRNA action may well be employed in HBV-related HCC, but this remains to be established. Not surprisingly, novel lncRNAs aberrantly expressed in HBV-related HCC tissues continue to be discovered. Future studies will undoubtedly shed more insight into their roles in promoting HBV-related HCC. LncRNAs may be useful as diagnostic or prognostic markers and could be considered as potential targets for the treatment of HBV-related HCC.

## Author Contributions

CS and NS contributed equally to this work by writing the review article and creating the figures. AE, KB, and PA reviewed and edited the manuscript. All authors approved the submitted version.

## Funding

Financial support from the South African National Research Foundation (Unique Grant Numbers: 118022 and 120383), The Poliomyelitis Research Foundation, and extramural unit baseline funding from the South African Medical Research Council is gratefully acknowledged.

## Conflict of Interest

The authors declare that the research was conducted in the absence of any commercial or financial relationships that could be construed as a potential conflict of interest.

## Publisher’s Note

All claims expressed in this article are solely those of the authors and do not necessarily represent those of their affiliated organizations, or those of the publisher, the editors and the reviewers. Any product that may be evaluated in this article, or claim that may be made by its manufacturer, is not guaranteed or endorsed by the publisher.
